# Human Ovarian Cancer Tumor Formation in Severe Combined Immunodeficient (SCID) Pigs

**DOI:** 10.3389/fonc.2019.00009

**Published:** 2019-01-22

**Authors:** Adeline N. Boettcher, Matti Kiupel, Malavika K. Adur, Emiliano Cocco, Alessandro D. Santin, Stefania Bellone, Sara E. Charley, Barbara Blanco-Fernandez, John I. Risinger, Jason W. Ross, Christopher K. Tuggle, Erik M. Shapiro

**Affiliations:** ^1^Department of Animal Science, Iowa State University, Ames, IA, United States; ^2^Department of Pathobiology and Diagnostic Investigation, College of Veterinary Medicine, Michigan State University, East Lansing, MI, United States; ^3^Department of Obstetrics, Gynecology and Reproductive Sciences, Yale University School of Medicine, New Haven, CT, United States; ^4^Human Oncology and Pathogenesis Program, Memorial Sloan Kettering Cancer Center, New York, NY, United States; ^5^Department of Radiology, Michigan State University, East Lansing, MI, United States; ^6^Department of Obstetrics, Gynecology and Reproductive Biology, Michigan State University, Grand Rapids, MI, United States; ^7^Institute for Quantitative Health Science and Engineering, Michigan State University, East Lansing, MI, United States

**Keywords:** ovarian cancer, severe combined immunodeficient, swine, preclinical animal model, Claudin

## Abstract

Ovarian cancer (OvCa) is the most lethal gynecologic malignancy, with two-thirds of patients having late-stage disease (II-IV) at diagnosis. Improved diagnosis and therapies are needed, yet preclinical animal models for ovarian cancer research have primarily been restricted to rodents, for data on which can fail to translate to the clinic. Thus, there is currently a need for a large animal OvCa model. Therefore, we sought to determine if pigs, being more similar to humans in terms of anatomy and physiology, would be a viable preclinical animal model for OvCa. We injected human OSPC-ARK1 cells, a chemotherapy-resistant primary ovarian serous papillary carcinoma cell line, into the neck muscle and ear tissue of four severe combined immune deficient (SCID) and two non-SCID pigs housed in novel biocontainment facilities to study the ability of human OvCa cells to form tumors in a xenotransplantation model. Tumors developed in ear tissue of three SCID pigs, while two SCID pigs developed tumors in neck tissue; no tumors were detected in non-SCID control pigs. All tumor masses were confirmed microscopically as ovarian carcinomas. The carcinomas in SCID pigs were morphologically similar to the original ovarian carcinoma and had the same immunohistochemical phenotype based on expression of Claudin 3, Claudin 4, Cytokeratin 7, p16, and EMA. Confirmation that OSPC-ARK1 cells form carcinomas in SCID pigs substantiates further development of orthotopic models of OvCa in pigs.

## Introduction

Ovarian cancer (OvCa) is the most lethal among gynecologic malignancies, taking an estimated 14,000 lives in the United States in 2018 (1). OvCa often goes undetected until late stages due to non-specificity of its early symptoms, hence 2/3 of patients have late-state disease (stage III–IV) at diagnosis. The current standard of care is debulking surgery to remove tumor masses followed by first-line platinum and Taxol chemotherapy (2). Debulking is critical to successful chemotherapy, and so prior identification of tumor masses by diagnostic imaging often plays a key role in pre-surgical planning. X-ray computer tomography (CT) (3) is the most widely used imaging modality for evaluating peritoneal spread in OvCa for presurgical planning, yet there are well acknowledged “blind-spots” where tumor spread simply cannot be seen as the contrast between normal tissue and tumors is insufficient to discriminate one tissue type from another. Thus, tumors can be missed, leading to incomplete tumor resection. Methods to enhance tumor detection are being developed for a variety of imaging modalities, including magnetic resonance imaging (MRI) and positron emission tomography (PET), making use of various targeting mechanisms to specifically target ovarian cancers. For example, small peptides and molecules including OTL38 (4), GE11 (5), and CPE (6–9), which bind to folate receptor, EGFR, and claudins, respectively, have been successfully tested in preclinical mouse trials. Clinical trials for use of OTL38 are already beginning to recruit patients with folate receptor positive ovarian cancer for the use of this fluorescent molecule during cytoreduction or debulking surgeries (10).

Current research to improve imaging technologies and methodologies uses either human volunteers or rodent models. Imaging research involving human OvCa patients is challenging for a variety of reasons. For imaging modalities which involve significant radiation, such as CT, extensive research cannot be performed due to radiation dose. Second, it is challenging to perform serial imaging research on OvCa patients due to ongoing treatment regimens and patient morbidity. Such serial scans could be useful in developing predictive imaging capability derived from a multiparametric data set (11). Development of imaging strategies on rodents poorly informs how one approaches the clinical scenario. This is due to the size of the animal and the tumors, as well as the equipment that are used in animal imaging experiments. Small tumors exhibit different perfusion (12) and water diffusion (13) from large tumors. Small animals have different metabolism than large animals and exhibit vastly different pharmacology of administered drugs (14), while humans and pigs have similar liver content of cytochrome proteins (15, 16). All of these characteristics, and many more, influence imaging results.

It is not always practical to use human OvCa patients for developing or validating new imaging techniques, and rodents are inadequate for developing clinically relevant imaging protocols. Recently there is a new hybrid field of molecular imaging and surgery called optical surgical navigation (17). This field couples fluorescence imaging with surgery to enhance surgical removal of tumors by way of fluorescent marker uptake. The translational value of new fluorescent tracers, either targeted or untargeted, can only be meaningfully evaluated in the context of a research subject that has appropriate size and physiology to OvCa patients. A pig model of OvCa could fill this crucial gap.

Human cancer xenotransplantation studies have not been possible in pigs until the recent identification (18) or creation (19–22) of severe combined immunodeficient (SCID) pigs (23). SCID pigs have previously been reported to accept grafts of human melanoma (A375SM) and pancreatic carcinoma (PANC-1) cancer cell lines (24), as well as human induced pluripotent stem cells (25). In addition to xenotransplantation methods of studying cancer, genetic models of porcine cancer have also been developed. Inducible and germline mutations of TP53^R167H^ (26, 27) and KRAS^G12D^ (28, 29) have been introduced into pigs, which are useful in studying lymphomas, osteogenic tumors, renal tumors, and others. These neoplasms were detected with computed tomography (CT), magnetic resonance imaging (MRI), and ultrasound imaging systems (27), exemplifying the pig's utility as an imaging animal model system.

To initiate the development of a large animal model of ovarian cancer, we tested whether human ovarian cancer cells could survive and develop ectopic tumors in SCID pigs. OSPC-ARK1 cells, derived from an ovarian serous papillary carcinoma (OSPC), were injected into male and female SCID pigs and were monitored for tumor development for this first stage screen. We demonstrate that OSPC-ARK1 derived carcinomas developed in three of four SCID pigs tested. Additionally, we verified an immunophenotype comparable to human patient OSPC samples based on the expression of Claudin 3, Claudin 4, Cytokeratin 7, p16, and EMA in SCID pig carcinomas. In summary, we demonstrate that SCID pigs can successfully develop OSPC-ARK1 carcinomas, which warrants further development of an orthotopic SCID pig model of ovarian cancer.

## Materials and Methods

### Generation and Care of Piglets

Wildtype and ART^−/−^ piglets were generated as described (18) and were housed in positive pressure biocontainment bubble facilities (30). All animal protocols and procedures were approved by Iowa State University's Institutional Animal Care and Use Committee (IACUC).

### Human Tissue Collection

Informed consent was obtained from human subjects and was approved by the Yale Institutional Review Board. The OSPC-ARK1 primary ovarian cell line used in this study was established from samples collected at the time of tumor recurrence from a patient harboring stage IV ovarian serous papillary carcinoma.

### Cell Preparation and Injections

#### SCID Pigs

OSPC-ARK1 cells were grown in complete RPMI media (10% FBS, 50 μg/mL gentamycin, 10 mM HEPES) until 80–100% confluent. Cells were trypsinized and washed five times in sterile phosphate buffered saline (PBS). Cells were then counted with a hemocytometer and were brought to a concentration of 50 × 10^6^ cells/mL.

Animals were anesthetized with isoflurane. A total of six pigs were used in two independent experiments. Injection sites were marked and all animals were injected subcutaneously in the right and left ear and intramuscularly into the right and left side of the neck. In the first trial, four 43 day old pigs (S1, S2, NS1, and NS2) were injected with 5 million cells in a 100 μL PBS cell suspension. In the second trial, two 18 day old SCID pigs (S3 and S4) were injected with the same number and volume of cells in the same locations. The SCIDs in trial 1 were female, while the SCIDs in trial 2 were male. Table 1 shows an overview of piglet ID, sex, trial, genotype, age at trial end, and locations of carcinoma formation.

**Table 1:** SCID and non-SCID pig descriptions and tumor growth locations.

**Pig ID**	**Sex**	**Trial #**	**Days on trial**	**Age at end of trial**	**R Ear**	**L Ear**	**R Neck**	**L Neck**
S1	F	1	13	56 d	+	–	+	–
S2	F	1	30	73 d	–	–	–	–
S3	M	2	11	29 d	+	+	+	+
S4	M	2	7	25 d	+	–	–	–
NS1	M	1	30	73 d	–	–	–	–
NS2	M	1	30	73 d	–	–	–	–

#### SCID Mice

C.B-17/SCID female mice 5–7 weeks old were purchased from Harlan Sprague-Dawley (Indianapolis, IN) and housed in a pathogen-free environment. They were given basal diet and water *ad libitum*. All animal protocols and procedures were approved by Yale University's IACUC. OPSC-ARK1, a chemotherapy-resistant primary ovarian serous papillary carcinoma cell line, was used to develop a xenograft models. OPSC-ARK1 cancer cell line was injected IP at a dose of 7 million cells.

### Tissue Collection and Processing

Tissues were collected at the marked injection sites and fixed in 10% neutral buffered formalin for 24 h, at which point they were transferred to 70% ethanol. Following routine processing, tissues were embedded into paraffin and serial sections were cut at 5 μm thickness. Slides were either stained with hematoxylin and eosin or used for immunohistochemical labeling. In addition, OPSC-ARK1 cells were harvested and fixed in 10% neutral buffered formalin for 24 h and transferred to 60% alcohol. Following centrifugation, cell pellets were harvested and suspended in liquid histogel (Thermo Fisher Scientific, Ann Arbor, MI, USA). After the histogel had solidified, samples were transferred to 60% alcohol followed by routine tissue processing and embedding into paraffin. Serial sections were cut at 5 μm thickness and processed in parallel to the tissue samples.

### Immunohistochemistry

Serial sections of the pig tumors and the cell line were routinely labeled with immunohistochemistry for Claudin 3, Claudin 4 (both Thermo Fisher Scientific, Ann Arbor, MI, USA), Cytokeratin 7 (Agilent, Santa Clara, CA, USA), p16 (BD Biosciences, Franklin Lakes, NJ, USA), and EMA (LifeSpan BioSciences, Seattle, WA, USA). In addition, OPSC-ARK1 xenotransplant tumors from mice and sections of the original biopsy of this neoplasm that had been processed in a similar manner as the pig tissues were run as controls. Immunohistochemistry was performed on the Dako link 48 Automated Staining System (Agilent Technologies, Santa Clara, CA, USA) using the peroxidase conjugated EnVision Polymer Detection System (Agilent Technologies, Santa Clara, CA, USA) for all antibodies. Briefly, endogenous peroxidases were neutralized with 3% hydrogen peroxide for 5 min. Antigen retrieval was achieved by incubating slides in either low pH (Claudin 3, Claudin 4 and EMA) or a high pH (EMA) retrieval solution for 20 min on the Dako PT link (Agilent Technologies, Santa Clara, CA, USA) or through 20 min of protein digestion with proteinase K (Cytokeratin 7). Non-specific immunoglobulin binding was blocked by incubation of slides for 10 min with a protein-blocking agent (Agilent Technologies, Santa Clara, CA, USA). Using the Dako autostainer, slides were incubated for 30 min with a rabbit polyclonal anti-human Claudin 3 antibody (#34-1700), a mouse monoclonal anti-human Claudin 4 antibody (clone 3E2C1), a mouse monoclonal anti-human Cytokeratin 7 antibody (clone OV-TL 12/30), a rabbit polyclonal anti-human EMA antibody (#LS-C30532) and a mouse monoclonal anti-human p16 antibody (clone G175-405) at dilutions of 1:100, 1:250, 1:75, 1:500, and 1:100, respectively. The immunoreactions were visualized with 3,3-diaminobenzidine substrate (Dako, Carpinteria, CA). Sections were counterstained with Mayer's haematoxylin.

## Results

### OSPC-ARK Cells Injected Into SCID Pigs Develop Carcinomas

To assess if human ovarian carcinomas could develop in SCID pigs, a total of four SCID (S1, S2, S3, and S4) and two non-SCID (NS1 and NS2) pigs were injected with OSPC-ARK1 cells. Due to limited female SCID availability, we injected two female and two male SCID pigs in this initial study to answer our question of if OSPC-ARK1 cells were capable of developing tumors ectopically in immunocompromised pigs. Four sites were injected in each pig; one subcutaneous on each ear, and one intramuscular on each side of the neck. We decided to inject in these superficial sites such that we could easily and noninvasively monitor tumor growth over time. Evidence of growth in these sites would warrant injection into a more physiologically relevant area, such as the peritoneum or ovary of female SCID pigs.

**Figure 1:**
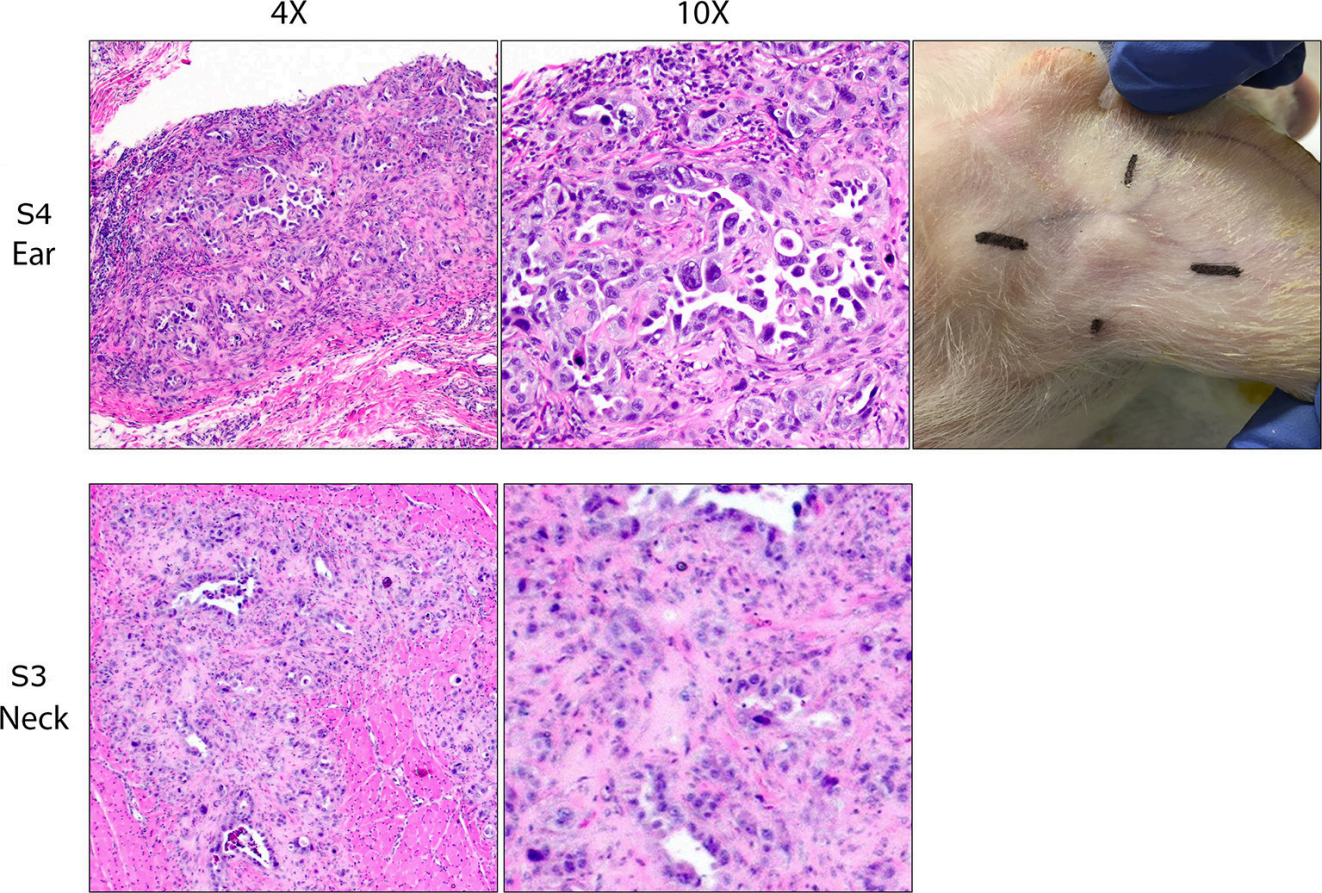
OSPC-ARK1 cells develop into carcinomas after subcutaneous and intramuscular injection in SCID pigs. SCID pigs were injected with OSPC-ARK1 cells subcutaneously in the ear and intramuscularly in the neck. Of the four SCID pigs, three developed carcinomas in the ear, and two developed carcinomas in the neck. H&E staining of carcinomas from the ear of S4 and neck of S3 are shown. Elongated cleft like glandular structures lined by anaplastic neoplastic cells and surrounded by a scirrhous response are a characteristic finding of high grade serous ovarian carcinomas and are easily recognizable in both the original human patient carcinoma and the carcinoma developing in the SCID pig.

Palpable tumors were observed on the ears of three SCID pigs prior to euthanasia. S1 and S2 were euthanized at 13 and 30 days after neoplastic cell inoculation and no grossly visible tumors were observed on the neck sites of injection. S3 was euthanized 11 days after injection and tumors were observed on the left and right ears. S4 was euthanized 7 days after injection, at which point a tumor was detected on the right ear (Figure 1). Wild-type animals, NS1 and NS2, did not have visible tumors at 30 days post injection. Table 1 shows an overview of locations of carcinoma development in the six pigs.

At euthanasia, samples from each injection site were collected and fixed, and H&E staining and analysis was used to determine if tumor architecture was present. Of the four SCID pigs injected, ovarian carcinomas were present in three animals in at least one injection site. S1 and S3 (Figure 1) developed carcinomas in the neck. S1, S3, and S4 all developed carcinomas within the ear tissue. S3 had carcinoma present in all four injection locations. In all cases neoplastic cells incited and were surrounded by an extensive scirrhous response. Most commonly, neoplastic cells formed small nests, solid cords or elongated cleft like glandular structures that were lined by anaplastic neoplastic cells. There was marked anisocytosis and anisokaryosis and the degree of cellular pleomorphism and the remarkable scirrhous response are characteristic findings of high grade serous ovarian carcinomas. In summary, we were able to demonstrate that OSPC-ARK1 cells were able to successfully form ovarian carcinomas in SCID pigs.

### OSPC-ARK1 Carcinomas in SCID Pigs Maintain Expression of Common Ovarian Carcinoma Diagnostic Markers

**Figure 2:**
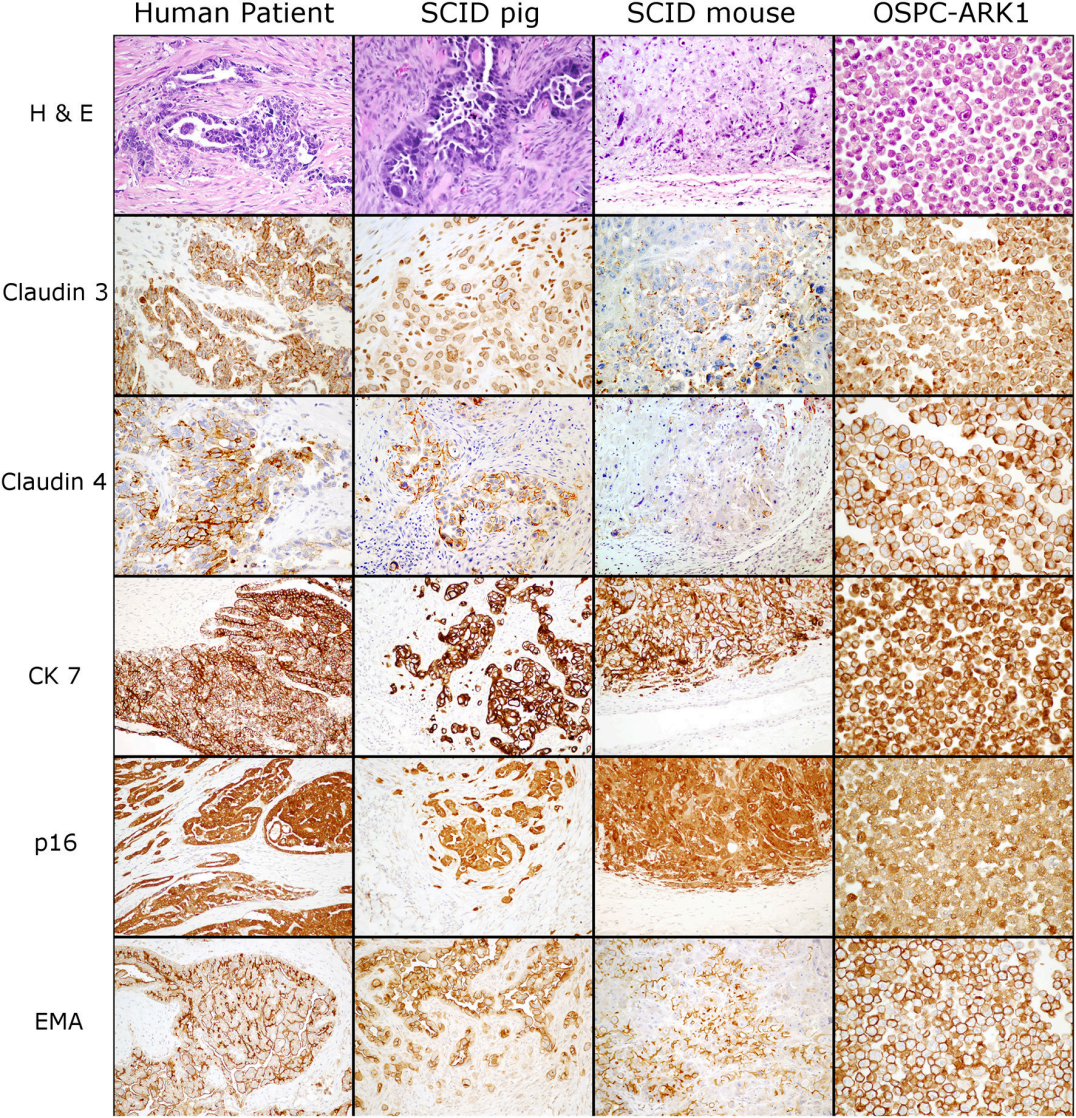
OSPC-ARK1 carcinomas in SCID pigs resemble the human ovarian serous papillary carcinoma morphologically and demonstrate the same immunophenotype. OSPC human patient ovarian carcinoma, OSPC-ARK1 carcinoma from S1, OSPC-ARK1 carcinoma from a SCID mouse, and OSPC-ARK1 neoplastic cells were stained with H&E and immunhistochemically labeled with Claudin 3, Claudin 4, CK7, p16, and EMA.

We next wanted to determine if ovarian carcinoma protein marker expression were retained in the pig xenotransplants. OSPC-ARK1 cells, the original human carcinoma (neoplasm from which the OSPC-ARK1 cell line was derived), and OSPC-ARK1 derived carcinomas in SCID pigs and SCID mice were subjected to immunohistochemical analysis (Figure 2). S1 (female) is shown in Figure 2. We assessed the expression of p16, epithelial membrane antigen (EMA), cytokeratin 7 (CK7), which have previously been used in diagnostic panels (31); we also assessed expression of Claudin 3 and 4 in all samples. Expression of CK7, p16, and EMA were all highly similar in tissue samples from all three species, as well as in the pellets generated from the OSPC-ARK1 cell line. Importantly, Claudin 3 and 4 expression in SCID pig carcinomas was also highly similar to the observed expression pattern in the original human carcinoma. In summary, OSPC-ARK1 carcinomas in SCID pigs have the same immunophenotype as the original ovarian carcinoma from a human patient.

## Discussion

We have described the successful development of human OSPC-ARK1 carcinomas in SCID pigs. Injected sites were verified histopathologically as true carcinomas. We additionally showed that tumors in SCID pigs phenotypically resembled human ovarian carcinomas through assessing the expression of OvCa protein markers CK7, p16, and EMA (31). Furthermore, we showed that tumors in SCID pigs retained expression of Claudin 3 and Claudin 4, which we have previously used as an imaging and therapeutic target in mouse models (6–8).

The ability of human ovarian tumors to grow in SCID pigs warrants further development of an orthotopic model of this cancer. The capability to study a human tumor in a non-rodent species is critically important as it would allow researchers and medical practitioners to utilize imaging modalities that are used in clinical settings. Additionally, there are many cases where progression from early-stage to late-stage occurs rapidly and can often happen within the span of a few months. We have raised SCID pigs for up to 6 months (unpublished results) in biocontainment facilities (30), which would allow long term trials that are required for studying spread and metastasis to be performed. The SCID pigs used in this study have natural mutations in *ARTEMIS* (18), which is a critical component of the VDJ recombination pathway required for TCR and BCR development, and thus have a T^−^ B^−^ NK^+^ cellular phenotype. NK cells are functional in our SCID model in *in vitro* assays (32), which could have anti-tumor activity on human cancer cells as evidenced by the absence of tumor development in S2. Thus, use of a T^−^ B^−^ NK^−^ SCID pig may better facilitate human ovarian tumor growth.

Pigs and humans share more similar reproductive tract sizes and structures than mice. In this initial trial, tumor growth from the OSPC-ARK1 cell line was not dependent on the sex of the animal. However, as we develop this model further, we would inject OSPC-ARK1 cells into the peritoneum or ovarian bursa in female SCID pigs. Such orthotopic tumor sites would allow for new imaging research to be initiated in SCID pigs. Studies involving surgical practices cannot be efficiently performed in mice because tumors are too small. Moving forward, it will be an important step to test human specific imaging targets (CPE, folate, GE11) and systems (PET, MRI) in SCID pigs xenografted with human tumors.

We have previously utilized the CPE peptide to either label tumors with a fluorescent marker (8) or deliver a suicide gene (9) to the site of ovarian tumors mice. Inoculation of OSPC-ARK1 cells into SCID pigs would allow for methods, dosages, efficacy, and safety of the CPE peptide to be established. Additionally, injection of fluorescently labeled CPE would allow for surgical practices to be performed for use of this peptide in marking smaller tumors that are difficult to detect by commonly used imaging practices. The pigs would be of comparable size to humans, so dosages of the peptide would be relevant as well. In all, confirming that human ovarian carcinomas can successfully develop in SCID pigs provides a basis for further development of an orthotopic OvCa model in pigs.

## Ethics Statement

This study was carried out in accordance with the recommendations of Yale University & Institutional Review Board with written informed consent from all subjects. All subjects gave written informed consent in accordance with the Declaration of Helsinki. The protocol was approved by the Yale University & Institutional Review Board. This study was carried out in accordance with the recommendations of ARRIVE and PREPARE guidelines recommended by the Institutional Animal Care and Use Committee. These protocols were approved by the Iowa State University and Yale University & Institutional Animal Care and Use Committee.

## Author Contributions

AB was involved in SCID pig cell injections, compiling data, and writing manuscript. MK performed histological and immunohistochemical analyses and provided histological and immunhistochemical descriptions. MA and JaR were involved in OSPC-ARK1 cell preparation. EC, AS, SB, and BB-F were involved with SCID mouse cell injections and human sample collection. SC was involved in SCID pig care and maintenance throughout the trials. JoR was involved in experimental design. CT injected cells into pigs and performed dissections. CT and ES designed experiment and reviewed data. All authors were involved in editing the manuscript.

### Conflict of Interest Statement

The authors declare that the research was conducted in the absence of any commercial or financial relationships that could be construed as a potential conflict of interest.
